# Seasonality and ambient temperature at time of conception in term-born individuals – influences on cardiovascular disease and obesity in adult life

**DOI:** 10.3402/ijch.v72i0.21466

**Published:** 2013-10-15

**Authors:** Nadja Schreier, Elena Moltchanova, Tom Forsén, Eero Kajantie, Johan G. Eriksson

**Affiliations:** 1 Department of Health Promotion and Chronic Disease Prevention, National Institute for Health and Welfare, Helsinki, Finland; 2 Folkhälsan Research Centre, Helsinki, Finland; 3 Department of Mathematics and Statistics, University of Canterbury, Christchurch, New Zealand; 4 Department of General Practice and Primary Health Care, University of Helsinki, Helsinki, Finland; 5 Vasa Central Hospital, Vasa, Finland; 6 Unit of General Practice, Helsinki University Central Hospital, Helsinki, Finland

**Keywords:** temperature, seasonality, obesity, hypertension, cardiovascular disease

## Abstract

**Background:**

The influence of environmental conditions early in life – including temperature and season – on health later in life has so far not attracted much attention.

**Objective:**

Using data from the Helsinki Birth Cohort Study of 13,345 men and women, the influence of temperature and season at month of conception on birth weight, and on cardiovascular diseases and obesity-related traits in later life was studied.

**Design:**

Linear regressions were fitted to examine the relationship between birth weight/obesity-related variables/hypertension and alternatively month of conception and average temperature of month of conception. The incidence of both coronary heart disease and cerebrovascular disease was assumed to follow a Weibull hazard model, and was modelled accordingly using survival analysis techniques.

**Results:**

In women, unusually cold temperatures at month of conception predicted lower body mass index (BMI) and fat percentage, and protected from obesity. Warmer temperatures at month of conception were associated with higher risk for hypertension. In men, warmer temperatures around conception predicted lower BMI. No seasonal influences were detected on obesity-related variables, nor were there seasonal or temperature mediated influences on birth weight, coronary heart disease or cerebrovascular disease observed.

**Conclusions:**

We suggest that ambient temperature has an influence on obesity-related outcomes and hypertension. This merits further study, also with regard to other health outcomes and from a global perspective.

The Developmental Origins of Health and Disease (DOHaD) hypothesis proposes that conditions present early in life have long-term consequences on later health. Traditionally nutritional status, growth, living conditions and maternal stress have been focused upon (1–4). Environmental conditions including season and ambient temperature have so far not attracted much attention.

However, season and temperature might be an important factor for health, potentially influencing hormonal and other adaptive responses. Already in 1941, C. A. Mills (5) reported that season of conception has an influence on mental and physical development of the human being, with differences in college matriculation, different onsets of the menses in girls, as well as differences in height and weight. Season has a major influence on the average outdoor temperature, day length, amount of precipitation, nutrition and pollution. Also, viral agents are commonly seasonal. Furthermore, temperature can reach extremes within the typical seasonal range, and it may cause additional health problems.

Low birth weight has been associated with seasonality and lower outdoor temperatures in many studies, as well as with warmer temperatures during pregnancy (6–9). However, these studies mainly included prematurely born subjects. Low birth weight in term-born subjects indicates intrauteral growth retardation/restriction, and the subjects are called small for gestational age (SGA). Studies on seasonality and temperature effects on term-born SGA individuals are relatively rare. Two independent studies associated low temperature in the midtrimester with low birth weight in term-born infants (6, 10).

While small body size at birth has repeatedly been associated with coronary heart disease and hypertension in adult life (11–16), the influences of temperature and season during pregnancy on health in adult life have not been widely studied. Phillips (17) studied the influence of season at birth on the risk of obesity in adult life and found an association between early exposure to cold and increased risk of obesity later in life. Seasonal effects on obesity in adult life were also reported in a Canadian study, where subjects born in winter/spring were more likely to be morbidly obese (BMI ≥40 kg/m^2^) (18). Other health outcomes have also been related to season of birth, for example, cerebrovascular disease with an elevated risk of death from subarachnoid haemorrhage in subjects born in the warm season between June and September (19).

Finland is one of the northernmost countries, with both maritime and continental influences. Due to the continental influence and the Gulfstream, average temperatures are slightly higher than in other countries of the same latitude. In the capital city of Helsinki – one of the southernmost cities of the country, located on the 60th latitude – the average winter temperature is around −5°C and the summer average about 16°C. The yearly temperature range of Helsinki is very broad, ranging between −34°C and +34°C. Due to large seasonal variation, the climate of Helsinki provides a good basis for assessing the influence of season and temperature on human health outcomes.

In this study, we examine the influence of temperature and season on birth weight, cardiovascular disease and obesity in adult life in term births, using month of conception as a reference.

## Subjects and materials

The Helsinki Birth Cohort Study includes 13,345 men and women, of whom 4,585 were born at the Helsinki City Maternity Hospital and 8,760 at the University Hospital in Helsinki between 1934 and 1944. They attended child welfare clinics in the city and were living in Finland in 1971 when a unique personal identification number had been allocated to each resident of the country (20). The cohort data include birth characteristics, such as length and weight at birth, parity and father's occupational status, as well as the date of the mother's last menstrual period. Using the personal identification number, these records were linked to the Death Registry and to the Hospital Discharge Registry to obtain information on coronary heart disease, corresponding to ICD8 and ICD9 codes 410-414, and ICD10 codes: I21-25, as well as on cerebrovascular disease (ICD8 and ICD9 codes 430-438 and ICD10 codes: I60-69). For hypertension, we used the data of subjects who were entitled to special reimbursement for antihypertensive medication from the Social Insurance Institution of Finland. In Finland, entitlement to special reimbursement is determined by a physician at the National Social Insurance Institution who assesses a clinician's statement based on set criteria.

From the register cohort consisting of 13,345 people, 2,003 individuals were randomly selected and invited for a clinical examination in the years 2001–2004, as previously described (21, 22). Height and weight were measured in light indoor clothing and without shoes. Body mass index (BMI) was calculated as weight (kg) divided by the square of height (m). Body composition was measured by bioelectrical impedance analysis (BIA) using the InBody 3.0 eight-polar tactile electrode system, Biospace Co., Ltd, Seoul, Korea. It measures lean body mass and percentage body fat by segmental multifrequency analyses separately for each limb and trunk. The maximal BMI was calculated from the height and the self-reported highest weight attained during adult life, with the exception of weight during pregnancy. Obesity was defined as BMI being over the threshold of 30 kg/m^2^.

Data on average monthly temperature for the years 1923–1944 were obtained from the Finnish meteorological institute weather station Kaisaniemi, which is in the centre of Helsinki (23).

## Statistical methods

A linear regression with adjustment for gestational age, sex, father's occupation and parity was fitted to examine the relationship between birth weight and alternatively month of conception and average temperature of month of conception.

Also, a linear regression with adjustment for gestational age, sex, father's occupation, parity, birth weight and age was fitted to examine the same relationship for BMI and body fat percentage. BMI was logged to normalize the distribution of regression residuals. The prevalence of hypertension and obesity was similarly modelled using logistic linear regression.

The incidence of both coronary heart disease and cerebrovascular disease was assumed to follow a Weibull hazard model, and it was modelled accordingly using survival analysis techniques.

In addition to treating monthly average temperature as a continuous variable, the following grouping approaches were also explored. (i) The overall temperature quartile variable was formed according to whether the average temperature of a month was below the first quartile of the observations during the years 1923–1944, above the third quartile, or between the first and the third quartile. It indicates, whether the average temperature of a specific month was cold, warm or in the middle compared to all monthly average measurements of the time series available from 1923 to 1944. Therefore, the month of January would generally fall in the coldest group, that is, below the first quartile. (ii) The month-specific temperature quartile variable was formed according to whether the average temperature of a specific month was below the first quartile of the observations of all the other observations of the same calendar month in the years 1923–1944, above the third quartile, or between the first and the third quartile. It indicates, if, for example, November of a specific year was unusually cold, compared to all other Novembers of the time series available from 1923 to 1944.

The model fit and statistical significance was assessed using ANOVA.

All of the analyses were performed using R-software base and survival package (24, 25).

## Results

**Fig. 1:**
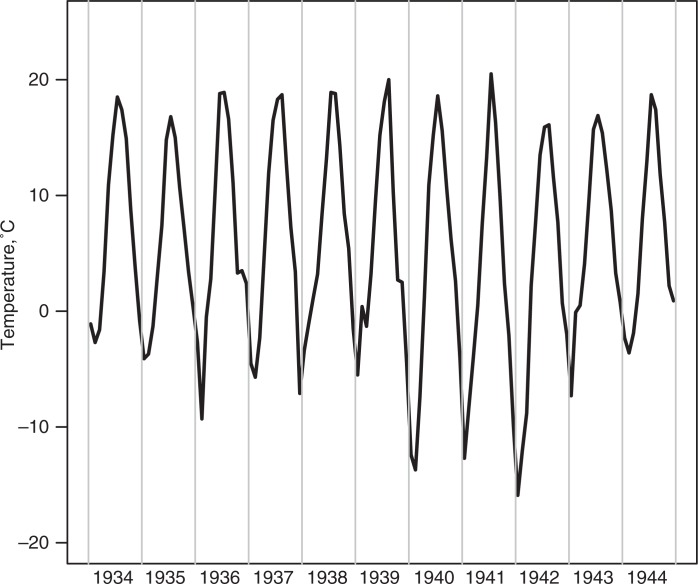
Monthly average temperatures in Helsinki, Finland in the years 1933–1945.

In the register-based sample (n = 13,345), we excluded 2,108 subjects due to incomplete data, and/or gestational ages below 37 complete weeks, the commonly used limit of preterm birth, or above 44 weeks, in which case the records were deemed unlikely to be correct. A total of 11,237 people with adequate data were included in the analysis on birth weight, coronary heart disease, hypertension and cerebrovascular disease. Fifty-two percent of the participants were males. In the clinical study sample (n = 2,003), we excluded 325 subjects, due to incomplete data, gestational ages below 37 completed weeks or above 44 weeks, and one because of an improbable high BMI >50. Eventually, the sample size was 1,678 with 46% males (Table I). The monthly temperature averages for the years 1934–1944 reached extremely cold temperatures in the winters of 1939/40, 1940/41 and 1941/42 with average temperatures in the coldest months between −13°C and −16°C, while summers appeared to be rather stable in temperature over the years (Fig. 1).

**Table I:** Characteristics of the clinical study and the register cohort samples

	Register cohort sample				Clinical study sample			
		
	Men		Women		Men		Women	
n	5,846		5,391		777		901	
Gestational age, weeks (mean, SD)	40.0	1.4	40.2	1.4	40.1	1.4	40.2	1.4
Birth weight, g (mean, SD)	3514.3	458.5	3370.3	433.7	3516.6	469.8	3373.8	438.1
Coronary heart disease, cases (number, %)	642	11	166	3	61	8	22	2
Cerebrovascular disease, cases (number, %)	323	6	170	3	36	5	18	2
Hypertension, cases (number, %)	1,161	20	1,019	19	186	24	188	21
BMI (kg/m^2^)					27.5	4	27.7	5
Obesity (number, %)					168	22	247	27
Fat percentage (%)					23.7	6	34.0	7
Parity (number, %)								
First child	2,780	48	2,504	46	372	48	419	47
Second child	1,686	29	1,627	30	209	27	287	32
Third child	785	13	703	13	111	14	117	13
Fourth or later child	595	10	557	10	83	11	78	9
SES at birth (number, %)								
1=higher official	744	13	603	11	102	13	88	10
2=lower official	1,270	22	1,241	23	158	20	168	19
3=labourer	3,832	66	3,547	66	517	67	645	72
Age-at-visit (years)					61.5	2.8	61.5	3.0

**Fig. 2:**
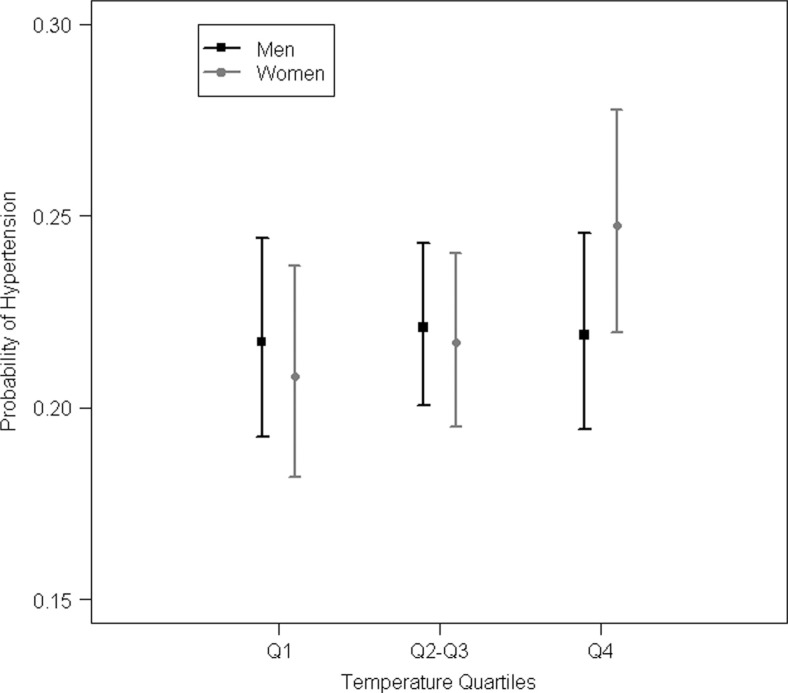
Hypertension risk by overall temperature quartiles of the month of conception, males and females, with 95% confidence intervals. The coldest monthly average temperatures are below the first quartile (Q1), while the warmest temperatures of our time series available are above the fourth quartile (Q4). (Q1: −16°C until −1.55°C, Q2–Q3: −1.56°C until 12.3°C, Q4: 12.31°C–21.5°C).

Warm temperatures around conception were found to be associated with a significantly higher probability of hypertension in women, that is, those who were conceived during the warmest temperatures of the time series from 1923 to 1944 (overall quartiles, register sample, Fig. 2, Table II). Hypertension was also generally associated with an increase of temperature in women (register sample). Furthermore, men exposed to an unusually warm month at conception time (month-specific quartiles) had a lower BMI in adult life (clinical sample). Birth weight, coronary heart disease and cardiovascular disease were not associated with warm temperatures at month of conception (register sample).

**Table II:** ANOVA p-values to assess the association between season/temperature and birth weight/coronary heart disease/cerebrovascular disease in the register cohort sample

	Season	Temperature (continuous)	TempQuart (overall)	TempQuart (month)
Men				
Birth weight	0.64	0.45	0.24	0.34
Coronary heart disease	0.59	0.26	0.22	0.90
Cerebrovascular disease	0.34	0.67	0.88	0.52
Hypertension	0.19	0.97	0.96	0.77
Women				
Birth weight	0.74	0.78	0.98	0.20
Coronary heart disease	0.49	0.25	0.08	0.36
Cerebrovascular disease	0.16	0.65	0.77	0.79
Hypertension	0.06	0.00*	0.04*	0.42

* Statistically significant at 5% level/TempQuart: temperature quartiles.

Cold temperatures around conception showed to have influences on obesity-related variables in women (clinical sample, Table III). Those women who were conceived during a month with average temperatures in the coldest quartile of this specific month over the time period of 1923–1944 (month-specific quartiles) had lower BMI, lower risk of obesity and lower fat percentage (clinical sample). Birth weight, coronary heart disease and cardiovascular disease were not associated with cold temperatures at the month of conception (register sample).

**Table III:** ANOVA p-values to assess the association between season/temperature and obesity variables in the clinical study sample.

	Season	Temperature (continuous)	TempQuart (overall)	TempQuart (month)
Men				
BMI	0.07	0.36	0.03*	0.63
Obesity	0.58	0.30	0.14	0.54
Fat percentage	0.65	0.24	0.29	0.41
Women				
BMI	0.63	0.84	0.66	0.02*
Obesity	0.41	0.82	0.87	0.02*
Fat percentage	0.77	0.75	0.77	0.03*

* Statistically significant at 5% level/TempQuart: temperature quartiles. Significant p-values indicate that the given factor has an effect on the respective outcome.

**Fig. 3:**
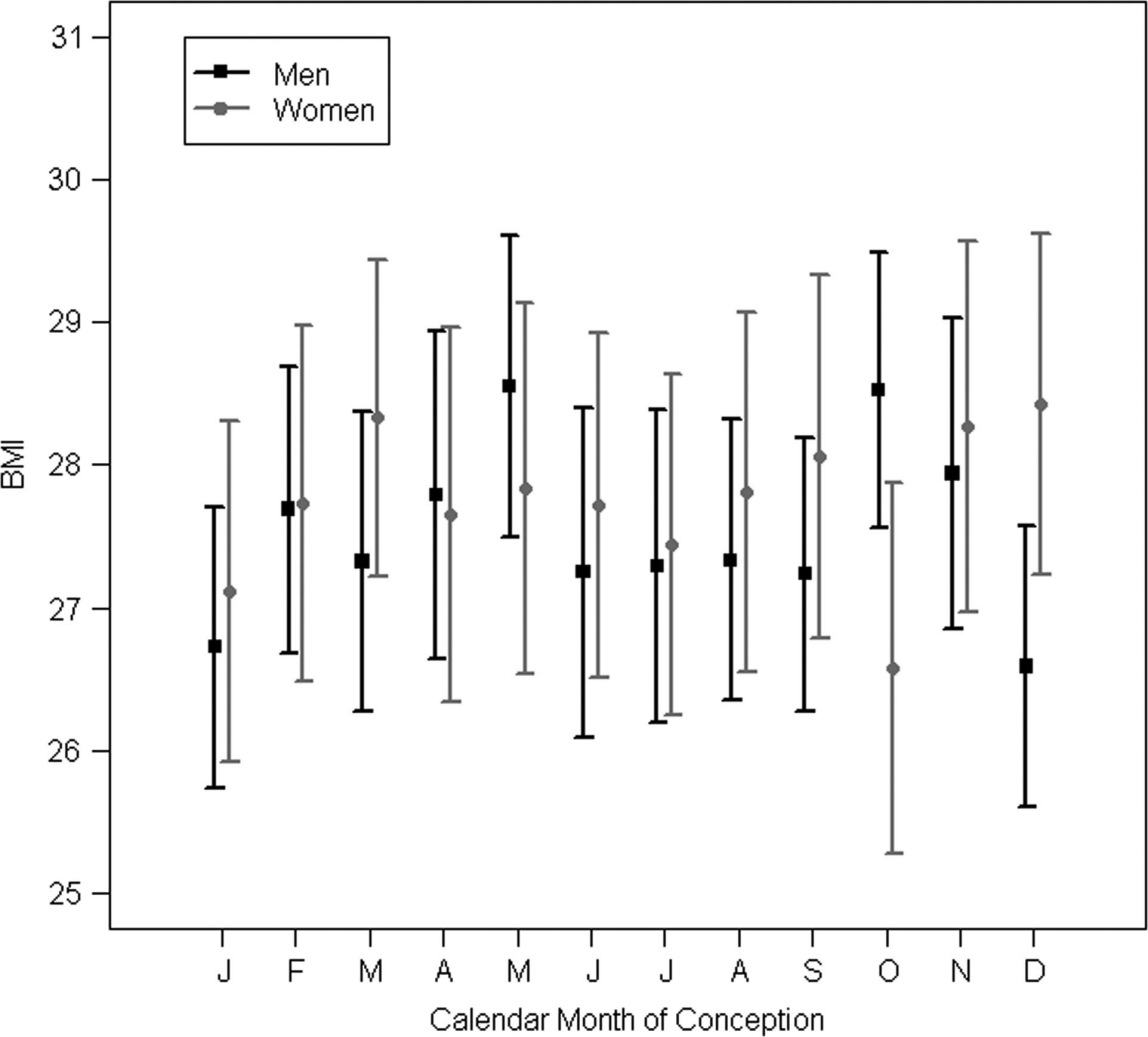
BMI by calendar month of conception, males and females, with 95% confidence intervals.

Season did not show any influences on the outcomes studied (Fig. 3).

## Discussion and conclusions

We studied the influences of season and temperature at month of conception on birth weight and later health outcomes in term-born subjects. In women, we observed protective influences on several obesity-related variables – BMI, obesity and fat percentage – among those conceived during unusually cold months. Furthermore, the risk of developing hypertension was enhanced with increasing temperatures at the time of conception. In men, warmer temperatures at month of conception were related to lower BMI. Our results suggest that temperatures at conception may have long-term influences on health outcomes.

We observed that women who were conceived during unusually cold months had lower values/risks for all obesity-related outcomes studied, that is, BMI, obesity and fat percentage. Unusual temperatures always require some form of adaptation, including a change of clothing, and depending on the season also a change in heating. Such adaptation usually requires time. Extremely cold temperatures, for example in February, would also lead to an increased need for heating and potential drops in indoor temperature are possible. This time of adjustment implies freezing, and therefore a potential lowering of the core temperature, which could have influences on the conceived embryo or on the selection of the sperm that fuses with the ovum. However, men who were conceived in unusually warm months had lower BMI. This association would need further investigation in which seasons these influences take place, as unusually warm winters differ essentially from unusually hot summers. The underlying mechanisms and explanations for these findings are unknown. Among people living in a region not used to colder temperatures it would mean that the bodies of the people are not acclimated to heat, and would therefore be easily overheated/stressed in unusually hot summers – also due to the fact that the inside temperature of the houses would rise unusually and not even cool down adequately at night due to the very well-insulated houses. A study in reptiles showed that temperature at conception can have significant impact on the offspring, by changing the sex ratio (26). A change in sex ratio can indicate a certain selection of the offspring, so that certain individuals with certain characteristics would be selected depending on the outdoor temperature. This could happen through the selection of sperm according to the temperature or through miscarriages of the “genetically weaker” embryos (27).

A study from Hertfordshire, UK, reported seasonality of BMI in men by birth month, with higher rates in January–June compared to the second half of the year (17). Furthermore, men born after relatively cold winters – using average temperatures of December and January with a cut-off point at 4°C as a reference – were more likely to have a higher BMI in adult life. In our analysis, season had no effect on the BMI in men. Furthermore, we could not detect any effects of cold months. However, our analyses were restricted to cold temperatures at conception and most probably have no influence on the temperature around birth. The UK study group did not find any effects in women, while we found lowering effects of unusually cold temperatures at the conception month on BMI. The methods of the two analyses were rather different, and therefore not easily comparable. Furthermore, the average temperatures in Helsinki of below 4°C would be from about November until April, compared to Hertfordshire from December until February.

Our study focused on term-born subjects. Potential impacts of prematurity and very low birth weight on life-long health have been discussed elsewhere (14, 28–30). There is a large body of evidence that birth weight is affected by season (7, 8, 31). However, most of those studies included preterm births. Due to the exclusion of preterm births in our study, the seasonal effect on birth weight might not be strong enough to reach statistical significance. Furthermore, the city of Helsinki is an urban area and might therefore not be as sensitive to seasonal variation as rural areas. Fallis and Hilditch (32), who compared rural and urban areas with respect to seasonal patterns of birth weight, found a stronger influence of season in rural areas. The proposed explanation is the higher variability of food intake in rural areas than in urban areas, in response to seasonal variation. However, wartime food restrictions were more likely to affect non-food-producing urban areas (33). Another aspect is that rural and urban areas differ in their occupational structures. Rural occupations, such as farming, usually include more frequent exposure to both cold and hot temperatures.

We did not find an influence of temperature extremes nor of seasonality on birth weight, despite the fact that Finland is a country with large temperature differences, and the winters of 1939/40–1941/42 were extremely cold. Temperatures in those years reached monthly average temperatures between −13°C and −16°C. Several studies on birth weight showed seasonal patterns indicating an influence of outdoor temperatures on the birth weight (6, 7, 9). The negative result in our study might be due to the harsh circumstances of the war – including severe stress and malnutrition, which could have such an effect that potential later health effects do not necessarily show through birth weight.

The study years of 1939–1944 were war years, involving severe stress and nutritional restrictions. In Helsinki, there was food rationing for many comestibles and the quality and quantity of the food was occasionally inadequate (34). However, the influence of nutritional restrictions would need extensive research, and could not be investigated in the framework of this study. Previous studies, such as the Dutch Hunger Winter study, the Channel Island Famine study and the Leningrad Siege study, studied the effect of prenatal malnutrition on later coronary heart disease and cardiovascular diseases, in general (35–38). The Dutch Hunger Winter study showed increased risks of coronary heart disease, hypertension, as well as type 2 diabetes in later life, while in the two other studies there were no associations found between malnutrition and cardiovascular disease or glucose intolerance. In our previous study of the effects of the bombings of Helsinki during World War II on subjects exposed while in utero, we observed slightly lower rates of both coronary heart disease and cerebrovascular disease in women, suggesting the effect of such a stress to be selective for developing these diseases if anything (4). In conclusion, the hardships of the war were multiple and their effect very hard to estimate. This is certainly a limitation of this study.

In this study, we used monthly average temperature measurements, as we did not have access to daily temperature measurements for all years. The analysis could be refined including daily temperatures, as the temperature extremes get smoothed using averages. However, we would not expect the results to change by including daily temperatures in the analysis. To our knowledge, there is no evidence to suggest that a few days of extreme temperatures would have an influence in utero on later health. Heat waves or cold spells which last for a week would certainly noticeably increase the monthly average, unless the same month contained an unusually cold week, which is a relatively improbable expectation. Furthermore, daily temperatures would add further challenges to the analysis, one is the high variation of the daily temperatures, and another is the necessity to include other parameters, for example, time lags.

In a pilot study, we have considered conditions and timing of pregnancy rather than the month of conception. However, we could not find any correlation with either season or temperature. Additionally, we used also month of birth as a reference point. However, in favour of better comprehensibility we decided to only use month of conception.

Our study examined the influences of season and temperature at month of conception on birth weight, coronary heart disease, cerebrovascular disease, hypertension, BMI, obesity and fat percentage in term births. The effect of temperature and season on the unborn in relation to adult health has not been focused upon in great detail previously. One reason might be the publication bias because of negative findings. We did observe temperature influences on all obesity-related variables as well as on hypertension in women and on BMI in men. There were no findings in relation to coronary heart disease and cerebrovascular disease nor were there any seasonal influences detected. It would be important to study this area in more detail – also on other health outcomes and in other regions of the world – as they are potentially important underlying factors. Furthermore, climate change with more extreme cold and extreme warm temperatures may exacerbate the influences of temperature on disease.

### Ethical considerations

The Ethical committees at Helsinki University Central Hospital and at the National Institute for Health and Welfare have approved the Helsinki Birth Cohort Study.
